# Microstructural Characteristics and Hardness Enhancement of Super Duplex Stainless Steel by Friction Stir Processing

**DOI:** 10.3390/ma15186267

**Published:** 2022-09-09

**Authors:** Linlin Pan, Chi Tat Kwok, Kin Ho Lo

**Affiliations:** 1 Guangdong Provincial Key Laboratory of Advanced Welding Technology, China-Ukraine Institute of Welding, Guangdong Academy of Sciences, Guangzhou 510650, China; 2 Department of Electromechanical Engineering, University of Macau, Macau 999078, China; 3 Institute of Applied Physics and Materials Engineering, University of Macau, Macau 999078, China

**Keywords:** friction stir processing, super duplex stainless steel, microstructural evolution, hardness

## Abstract

In the present study, microstructural evolution and hardness of the friction stir processed (FSPed) SAF 2507 super duplex stainless steel fabricated at a rotational speed of 650 rpm and a traverse speed of 60 mm/min were investigated. A scanning electron microscope (SEM) equipped with an electron backscatter diffraction (EBSD) detector was used to study the microstructure of the stir zone. The grain sizes of austenite and ferrite in the FSPed 2507 were found to be smaller (0.75 and 0.96 μm) than those of the substrate (6.6 and 5.6 μm) attributed to the occurrence of continuous dynamic recrystallization (CDRX) in both phases. Higher degree of grain refinement and DRX were obtained at the advancing side of the FSPed specimens due to higher strain and temperature. A non-uniform hardness distribution was observed along the longitudinal direction of the SZ. The maximum hardness was obtained at the bottom (407 HV_1_).

## 1. Introduction

Super duplex stainless steels (SDSSs) have higher molybdenum and chromium contents and a mixture of approximately equal fractions of austenite (γ) and ferrite (α), giving a combination of higher mechanical strength and corrosion resistance as compared with either austenitic or ferritic stainless steels [1]. SDSSs have become the alternatives to super austenitic stainless steels and Ni-based alloys because of their lower Ni content and price. SDSSs are widely used in many industrial fields including pulp and paper, oil, petrochemical, power generation, and pollution control industries [2].

Friction stir welding (FSW) is an alternative method in terms of overcoming the above problems as the welding temperature is relatively low. This solid-state welding method was invented at TWI in 1991 [3]. Recently, a new technology called friction stir processing (FSP) was developed by the basic principle of FSW [4] for various purposes, such as grain refinement [5], surface modification or hardening [6], and microstructural homogeneity [7,8]. In FSP, a consumable rotating tool is plunged into a workpiece and moves along the required regions. The severe plastic deformation coupled with high temperature and pressure during FSP would result in contamination and tool wear [9]. Translation of the rotating tool creates a characteristic asymmetry at the two sides of the processed zone, one is the advancing side (AS), where the direction of the tool rotation is the same as that of the tool translation, and the other one is the retreating side (RS), where the direction of the tool rotation is opposite to that of the tool translation [4].

Up to now, much progress has been made in FSW/FSP of duplex stainless steels by some researchers. Defect-free welds/surfaces can be achieved on duplex stainless steels by applying FSW/FSP [10,11,12,13]. Sato et al. [11] studied the microstructure and mechanical properties of FSWed SAF 2507. They reported that FSW could significantly refine the α and γ phases through dynamic recrystallization, the smaller grain size could lead to higher hardness and strength in the stir zone (SZ). Furthermore, multi-passes could make a further reduction in grain size of SAF 2507 after FSP [14]. Saeid and his co-workers [12,13] reported that continuous dynamic recrystallization (CDRX) in α and CDRX together with static recrystallization (SRX) in γ were the possible mechanisms of grain refinement in SZ of SAF 2205. The corrosion resistance of the FSWed hyper duplex stainless steel was improved by the fine grain structures [15].

Avila and his co-workers reported that there was a slight difference in microstructures in AS and RS of the friction stir processed zone as the cooling rate, peak temperature, and dwell time in AS were higher than those of RS [16]. As a result, the variation in the microstructure could lead to a difference in mechanical properties. In the previous work of the present authors, we found that the heat generation was different in AS and RS of FSPed 440C martensitic stainless steel resulting in non-uniform hardness distribution [17]. Furthermore, the difference in microstructure and properties of AS and RS of the FSPed specimens became more significant as the traverse speed increased [18]. Similarly, the existing temperature gradient along the longitudinal direction of the SZ would cause an inhomogeneous microstructure between the top and bottom regions. Mao and his co-workers reported that the gradient microstructure and mechanical properties were found in different positions along the depth of the nugget zone of the FSWed 7075 aluminum alloy (20 mm thick) [19]. The maximum hardness (132 HV) of the nugget (3 mm beneath the top surface) was found to be slightly lower than that of the base material (165 HV). Cao and co-workers conducted FSW on a 4 mm thickness SDSS plate [20]. The temperature at the top region reached 1074 °C, while the temperature at the bottom region was only 627 °C, and the temperature difference became more obvious at a higher rotation speed.

In summary, the microstructure of the entire SZ is heterogeneous and varies with the temperature and strain. Although some efforts have been conducted on the microstructural evolution and mechanical properties of DSS [12] and SDSS [11], the microstructural analyses at different locations (AS, RS, and regions along the longitudinal direction of SZ) of the FSPed SDSS are inadequate. Thus, in the present study, how the temperature and strain affect the microstructure in different regions (AS, center, RS, and along the depth of the SZ) of the SZ was systematically investigated. The relationship between the heterogeneity of microstructure and the hardness was studied aiming at a full understanding of the entire SZ.

## 2. Materials and Methods

### 2.1. Materials

The base material used in this study was UNS S32750 (SAF 2507) SDSS plates. The nominal compositions in weight percent are listed in Table 1. Multipass hot-rolling of the 20 mm slabs of SAF 2507 was conducted using a hot-rolling mill. The slabs were soaked at 1150 °C for half an hour followed by 4-pass hot-rolling processes. After each rolling step, the hot-rolled samples were reheated to 1150 °C in order to perform the next rolling step. To retain the high-temperature microstructure, the hot-rolled samples were rapidly quenched in water after the 4-pass hot-rolling processes. The final thickness of SAF 2507 was 5 mm. The total strain (thickness reduction) after the 4-pass rolling processes was 75%. The surface of the plates with dimensions of 100 mm × 200 mm × 5 mm was ground with 600-grit emery paper and then rinsed with ethanol for removing the oil before FSP.

### 2.2. Friction Stir Processing (FSP)

A friction stir welding machine (FSW-TS-M16, China FSW Center) was applied for FSP of the SAF 2507 SDSS plate. FSP was conducted at a traverse speed of 60 mm/min and a rotation speed of 650 rpm and the specimen was designated as FSP650. The stirring tool was made of a tungsten–rhenium alloy (W–25 wt.% Re) with a conical pin and a shoulder. The diameters of the shoulder, pin base, pin tip, and pin height were 12 mm, 4.5 mm, 3.5 mm, and 3 mm, respectively. In order to avoid surface oxidation of the specimens, argon was used as the shielding gas at a rate of flow of 15 L·min^−1^.

### 2.3. Metallographic and Microstructural Studies

The cross-sections of the FSPed specimens were mechanically ground and polished with 9, 3, and 1 μm diamond suspensions, and finally polished by 0.02 μm colloidal silica polishing suspension. The specimens were then electrochemically etched with the 10 M NaOH at 6 V for microstructural analysis using an optical microscope (OM, DMI3000M, Leica, Shangai, China) and a scanning electron microscope (SEM, Zigma, Zeiss) with an EDS detector (X-Max, Oxford Instrument, Abingdon, UK) and an EBSD detector (NordlysNano, Oxford Instrument). The EBSD was operated at 20 kV under a step size of 0.1 μm. The grain sizes of the two phases were analyzed using the HKL Technology channel 5 software according to the phase map. The grains were detected depending on the grain boundaries. All boundary segments with an angle higher than 10° were considered grain boundaries. The distribution of misorientation angle was analyzed using software (Tango) with the EBSD raw data taken from the FSPed specimens. Grain boundaries with misorientation exceeding 15° were defined as high angle boundaries (HABs) and misorientation between 3 and 15° was low angle boundaries (LABs). The sum of the fractions (%) of LABs and HABs should be equal to 100%. Twin boundaries (TBs) are characterized by a 60° misorientation angle at a rotation axis of <111>. TBs should be included in HABs.

The recrystallized fraction component in Tango was applied to detect the deformed, substructured, and recrystallized grains. The component in EBSD was used to measure the internal average misorientation angle within each grain. When the internal average misorientation of a grain was larger than the minimum angle (*θ_c_* = 3°), the grain was classified as deformed grain (in red). As the average grain misorientation is smaller than *θ_c_*, but the misorientation from subgrain to subgrain was above *θ_c_*, the grain was defined as substructured grain (in yellow). The remaining grains were classified as recrystallized grains (in blue).

The kernel average misorientation (KAM) was measured for describing the distribution of residual strains following a rainbow color scheme. Blue and red represented the minimum and maximum residual strains, respectively. The average misorientation was calculated between every pixel and its surrounding pixels, and the mean value was assigned to that pixel. In addition, misorientations exceeding 5° were discarded, so the misorientations associated with discrete subgrain and grain boundaries were excluded.

### 2.4. Hardness Test

Vickers hardness test was conducted using an automatic Vickers hardness analyzer (Wilson VH3100, Lake Blu, IL, USA) with a diamond indenter subjected to a 1 kg load, according to ASTM-E384 (2011). Before the test, the surface of the specimen was ground with 1000-grit emery paper and then cleaned with ethanol. The hardness test was carried out along the cross-section starting 0.1 mm beneath the surface and repeated at least 3 times.

## 3. Results

### 3.1. Metallographical Analysis

**Figure 1:**
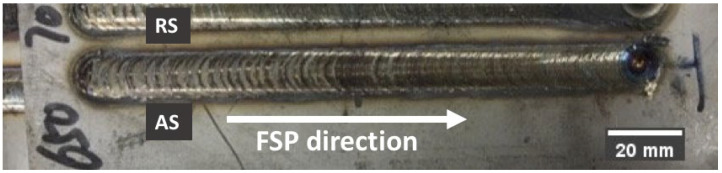
Top view of FSPed SAF 2507.

Figure 1 illustrates the macroscopic top view of the FSPed specimen (FSP650) with the AS, center, and RS of the SZ.

**Figure 2:**
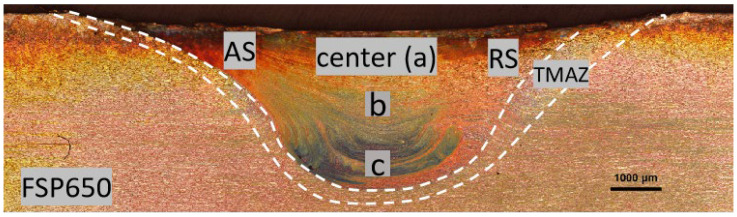
Cross-sectional view of the FSPed SAF 2507 taken with an optical microscope.

Figure 2 illustrates the cross-sectional view of SZ of FSP650 fabricated at a rotation speed of 650 rpm and a traverse speed of 60 mm/min without defects. Due to frictional heating and the severe plastic deformation during FSP, the SZ exhibited a basin-like shape [3]. The SZ is asymmetric due to different thermo-mechanical conditions at the AS, center, and RS. According to the work of Avila et al. [16], the temperature at the AS was found to be higher than that at the RS due to the high relative velocities at both sides of the rotating pin. In addition, the boundary between the thermo-mechanically affected zone (TMAZ) and SZ is clear at the AS, but the one at the RS is indistinct. It was reported that the distinct boundaries mainly depended on the viscosity gradient between the processed region and unprocessed region [21]. Higher temperature at the AS would result in a steeper viscosity gradient. Therefore, the boundary observed at the AS is sharp and distinct while the one at the RS is diffused. Sato and his co-workers investigated the microstructure and mechanical properties of the FSPed SAF 2507 SDSS [11]. They found that the AS experienced the most severe deformation because the translation of the stirring tool and tangential component of rotation were in the same direction resulting in larger frictional force. In addition, no obvious heat-affected zone (HAZ) was found in the FSPed specimens indicating that the growth of parent grains could be inhibited [22] because of low processing temperature and high cooling rate during FSP [23,24]. The microstructural evolution of three distinct regions (AS, center, and RS) will be analyzed in the next section.

### 3.2. Microstructural Analysis

#### 3.2.1. Different Regions along the Traverse Direction (AS, Center and RS)

The volume fractions of austenite and ferrite in different regions including the substrate (SUB), AS, center, and RS are summarized in Table 2. The locations of 0.1 mm beneath the processed surface of AS, center, and RS were selected. The volume fractions of austenite and ferrite of the SUB are 55% and 45%, respectively. The ferrite content of the center of the SZ is 50.7%, and is slightly higher than that of the SUB. This is attributed to the ferrite being more stable than the austenite when exposed to high temperature during FSP [11].

**Figure 3:**
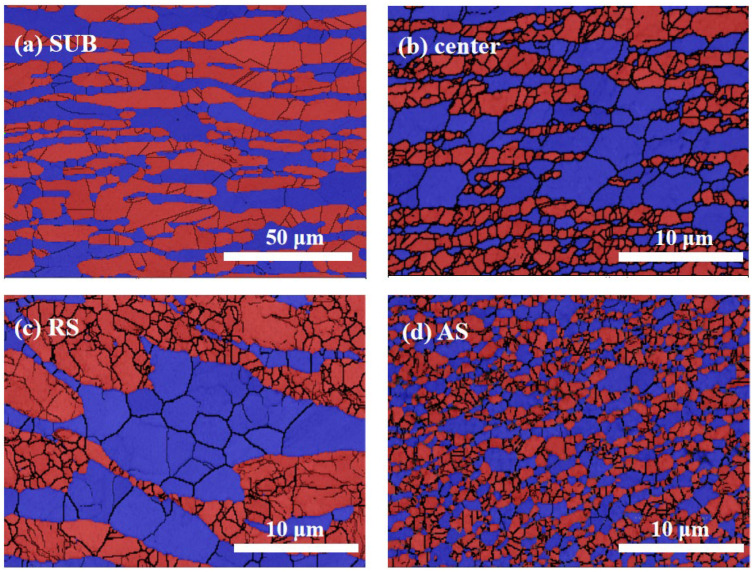
Phase maps of (**a**) SUB, (**b**) center, (**c**) RS, and (**d**) AS (austenite and ferrite are in red and blue respectively). Note that the scale bars in (**a**–**d**) are different.

**Figure 4:**
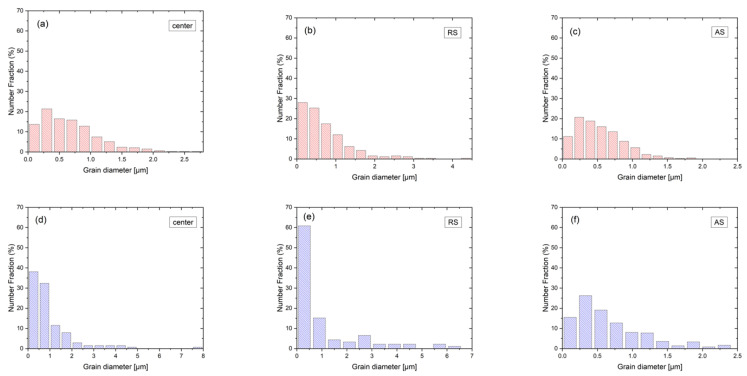
Phase maps of (**a**,**d**) center, (**b**,**e**) RS, and (**c**,**f**) AS (The grain sizes of austenite and ferrite bars are in red and blue, respectively).

The phase maps of the SUB and center are shown in Figure 3. The grain sizes were measured using the software Tango from the phase maps. The grain size distributions for center, RS, and AS are depicted in Figure 4. The SUB contains elongated ferrite and austenite grains with an average grain size of 5.6 and 6.6 μm, respectively. The microstructure of the center consists of a ferritic matrix with the austenite islands (Figure 3b). The average grain sizes of the ferrite and austenite in the center are 1.0 and 0.8 μm, respectively. The grain size is found to be reduced significantly after FSP because of dynamic recrystallization (DRX) [15]. In addition, no intermetallic phases were observed.

**Figure 5:**
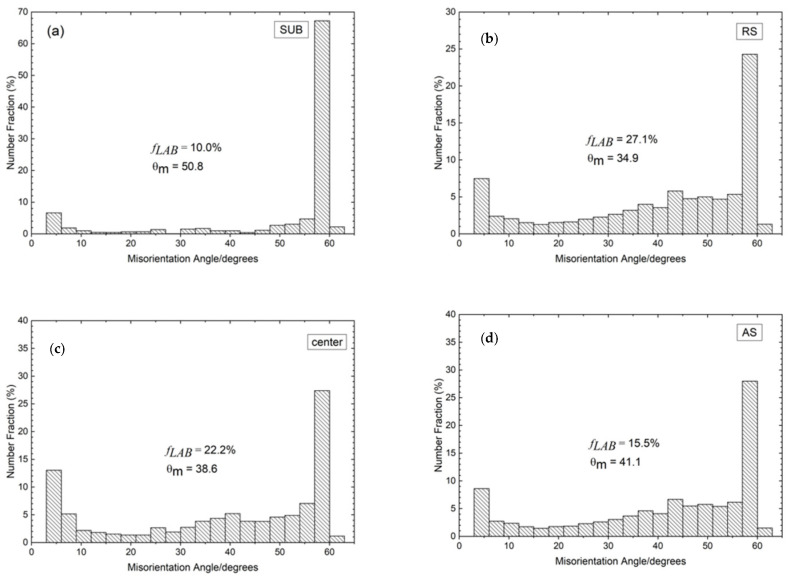
Misorientation angle histogram for γ/γ boundaries: (**a**) SUB, (**b**) RS, (**c**) center, and (**d**) AS (*f_LAB_*: fraction of low-angle boundaries, *θ_m_*: mean misorientation angle).

The misorientation angle histogram for the γ/γ boundaries of the SUB (SAF 2507) is shown in Figure 5. The distribution of misorientation in different regions of the FSPed specimen is similar to the SUB. In the SUB, there is a high fraction of HABs, while the fraction of LABs is relatively low (10.0%), as illustrated in Figure 5a. In addition, about 87.5% of HABs were a Σ3 twin relationship (60° rotation about <111> axis). The high fraction of twin boundaries originated from the growth of recrystallized grains in the austenite, which was reported by Mirzadeh et al. [25].

From Figure 5, the SUB contained the lowest amount of γ/γ LABs as compared to the FSPed specimens. On the contrary, the portion of twin boundaries (with misorientation angle = 60°) decreased from 70% to below 30% after FSP. The conversion of twin boundaries to the HABs was attributed to the intense deformation associated with FSP leading to complex interactions of twin boundaries with slip dislocations [13].

**Figure 6:**
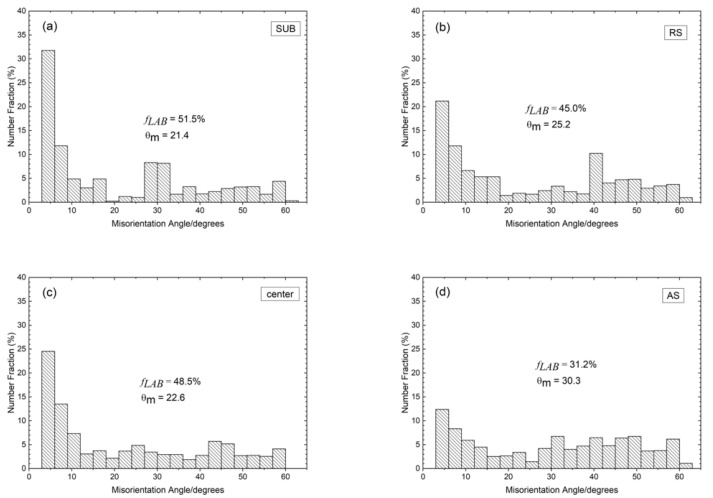
Misorientation angle histogram for α/α boundaries: (**a**) SUB, (**b**) RS, (**c**) center, and (**d**) AS.

Figure 6 depicts the misorientation distribution histogram for α/α boundaries of the SUB and different regions of the FSPed specimen. The distribution of misorientation of the different regions of the FSPed specimen is very different from that of the SUB. The ferrite in the SUB contains a considerable amount of LABs (51.5%) as shown in Figure 5a. The large number of LABs is probably derived from the initial rolled structure of the SUB [26]. There is a lower *f_LAB_* of AS (31.2%) as compared to those of center (48.5%) and RS (45.0%) as shown in Figure 5b–d. This phenomenon is related to different degrees of DRX of the three distinct regions, and hence different levels of evolution from LABs to HABs.

The formation mechanisms of grain structure during FSW of DSSs have been reported in the literature [11,12,13,15,27], and several restoration processes of DRX, dynamic recovery (DRV), and SRX could occur during FSW of the DSSs. It is well known that there are two types of DRX including discontinuous DRX (DDRX), which commonly occurs in stainless steels with low or medium stacking fault energy (SFE) by grain boundary migration; and continuous DRX (CDRX) which always takes place in the high SFE alloys by continuous fragmentation of substructure to form crystallites bounded with HABs [28]. Consequently, CDRX probably occurs in ferrite with higher SFE, whereas DDRX predominant occurs in austenite with a lower SFE. However, it has been reported that the DRX behavior of austenite was changed (DDRX → CDRX+SRX) in the DSS due to the co-existence of austenite and ferrite [12].

**Figure 7:**
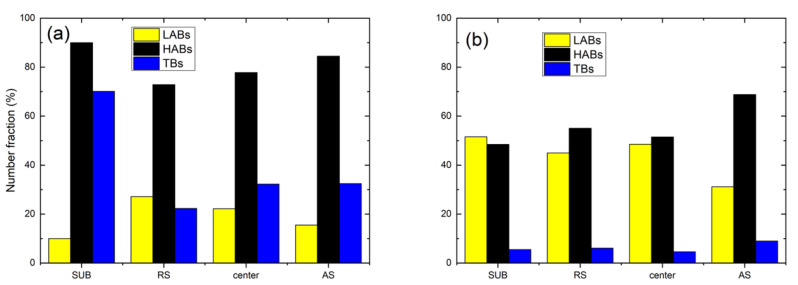
Grain boundary character distribution of SUB, RS, center, and AS: (**a**) γ/γ boundaries and (**b**) α/α boundaries (TBs: twin boundaries).

The grain boundary character distribution (GBCD) of different regions of the FSPed specimens is summarized in Figure 7. The *f_LAB_* in the austenite increased after FSP (Figure 7a). In the first stage, the formation of LABs in the austenite is attributed to the DRV during deformation by rearrangement of accumulating lattice dislocations [12]. Then the LABs transformed into HABs through absorption of mobile dislocations in the pre-existing boundaries because of the occurrence of CDRX. It was evidenced by the lower *f_LAB_* in AS and higher values in center and RS. Furthermore, the number of LABs in the ferrite decreased in the FSPed specimen as compared to that of SUB, which also revealed the mechanism of CDRX.

An increasing trend of a fraction of HABs and TBs from RS towards AS in both the two phases is observed in Figure 7. The variation of the misorientation distribution is mainly dependent on the temperature and degree of deformation [13]. It was reported that the temperature was the highest at the AS among all the processed regions due to the largest frictional force at the AS [16,29]. During CDRX, higher temperatures would promote the formation of HABs through thermally activated phenomena including cross-slip, dislocation movement, and climb [13]. On the other hand, the highest extent of deformation was generated at the AS and gave rise to more stored energy. Consequently, more LABs transformed to HABs through CDRX by absorbing dislocations [26].

Compared with the SUB, the grain sizes of RS, center, and AS were finer. It is because the FSPed specimens experienced severe deformation and frictional heat which induces the occurrence of DRX [3]. The grain size of austenite is smaller than that of ferrite in different regions of the FSPed specimen. It is well known that DRX easily occurs in the low-SFE phase, i.e., austenite [30]. On the other hand, DRX is hard to take place in the ferrite with high SFE. Conversely, the ferrite is more likely to undergo DRX than the austenite in duplex stainless steels. This is due to a priority accommodation of strain in ferrite and a higher diffusion rate of atoms in the ferrite as compared with the austenite at high temperatures [31]. Therefore, the recrystallized ferrite grains grow earlier than that of austenite grains after DRX.

In the SZ, the grain sizes of the austenite and ferrite decreased from the RS to the AS. Humphreys and Hatherly [28] reported that the growth of the recrystallized grains is limited by work hardening in the grain interior. Guerra et al. [32] proposed that the AS underwent the most severe deformation which would induce finer grain size. This is consistent with the present finding, i.e., the grain sizes of the austenite and ferrite at the AS were the finest as compared with those at the center and RS.

**Figure 8:**
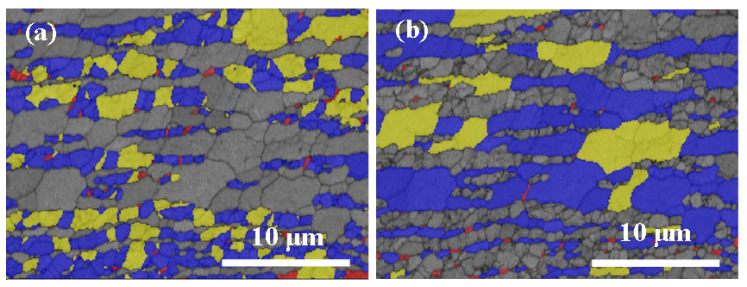
Recrystallized fraction (RF) maps of the center: (**a**) austenite and (**b**) ferrite (fully recrystallized grains are shown in blue, the deformed grains and the sub-structured grains are in red and yellow, respectively).

Figure 8 shows the typical recrystallized fraction (RF) maps at the center of the FSPed specimen. The number of recrystallized grains at the center was the largest among the different regions suggesting the occurrence of DRX in both phases during FSP. The appearances of the maps of AS, RS, and center were almost the same except for the fraction of recrystallized fraction. The deformed structure was surrounded by recrystallized grains in both austenite and ferrite in the SZ of the FSPed specimen (Figure 8). This implies that the recrystallized grains that grew by consuming the deformed structure are mainly attributed to the high diving force of nucleation and high dislocation density in the deformed regions [33].

**Figure 9:**
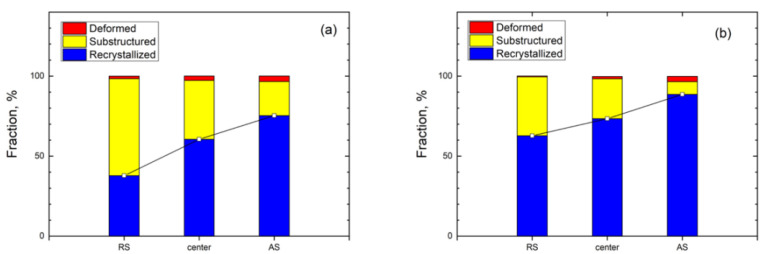
The fraction of the recrystallized, substructured, deformed grains at different locations of FSPed specimen: (**a**) austenite and (**b**) ferrite.

The fraction of the recrystallized, substructured, and deformed grains at different locations of the FSPed specimen is summarized in Figure 9. The number of recrystallized grains was the largest in different regions of the FSPed specimen compared to the deformed grains and the sub-structured grains suggesting dynamic recrystallization occurred in both phases during FSP.

The recrystallization process varied in different regions of the FSPed specimen because of the different temperatures and degrees of deformation in these areas. From Figure 9, the fraction of recrystallized and deformed grains presents a rising trend from the RS to AS. The increasing trend is in good agreement with the trend with LABs from RS to AS [25]. As mentioned above, the stored energy in AS was higher than that in RS, so it provided a larger driving force resulting in a higher degree of recrystallization [34]. In addition, the fraction of deformed grains shows an increasing trend from the RS to AS which is attributed to the higher degree of deformation generated at the AS.

It is observed that the fraction of recrystallized grains in the ferrite is higher than that of the austenite. The higher fraction of recrystallized grains in the ferrite was attributed to the strain accommodated in the ferrite. It was then transformed to the austenite resulting in a prior occurrence of DRX in the ferrite as compared with the austenite. Furthermore, the recrystallization process was nearly complete in the ferrite at the AS as the recrystallization fraction was about 90%.

#### 3.2.2. Different Regions along the Centerline

**Figure 10:**
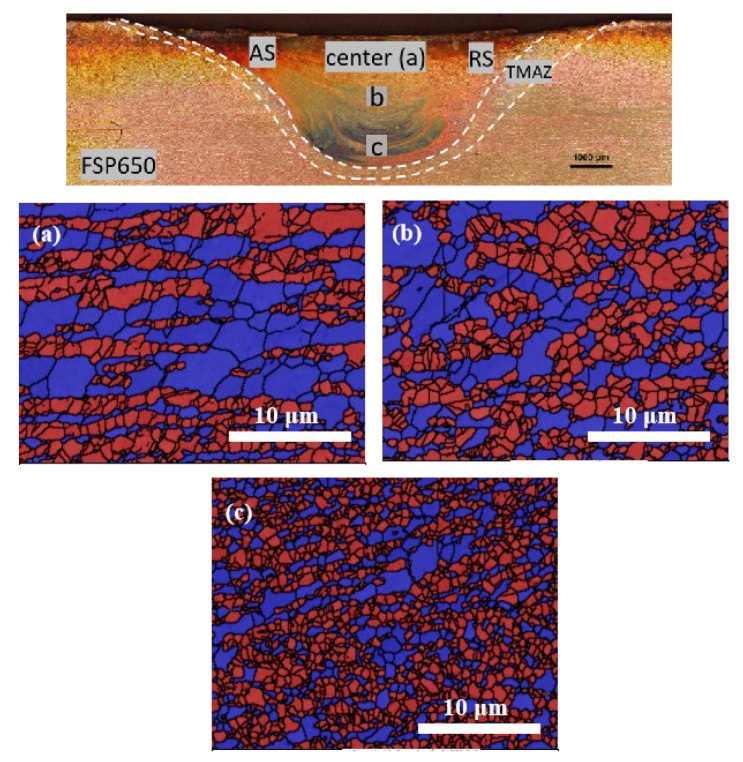
Phase maps of different regions (**a**–**c**) along the centerline of the FSPed SAF 2507 (ferrite and austenite are in blue and red, respectively).

Phase maps of the FSPed specimen from different regions (a, b, and c) along the vertical centerline are illustrated in Figure 10. Regions a, b, and c are 0.1, 1.5, and 2.9 mm beneath the surface, respectively. All the regions consist of the islands of austenite in the ferritic matrix and the microstructure is similar to that of the substrate. Region c has more refined austenite islands than regions a and b. The grain sizes of both austenite and ferrite decrease from the top to the bottom of the SZ along the centerline.

The volume fractions of austenite and ferrite are summarized in Table 3. The volume fraction of austenite increases with the depth along the centerline (Figure 10a–c). The variation in the relative content of the two phases was dominantly affected by the temperature achieved during FSP.

According to pseudo-binary Fe-Cr-Ni phase diagram for 70 wt% Fe [35], the temperature is estimated to be 1130 °C according to the phase fraction in region a (austenite: 49.3%, ferrite: 50.7%), which is close to the transformation temperature of γ to α (1125 °C). The temperature in region c is around 950 °C, which was the lowest among the three regions. In summary, the volume fraction of austenite increased with the decrease in temperature during FSP along the centerline.

**Figure 11:**
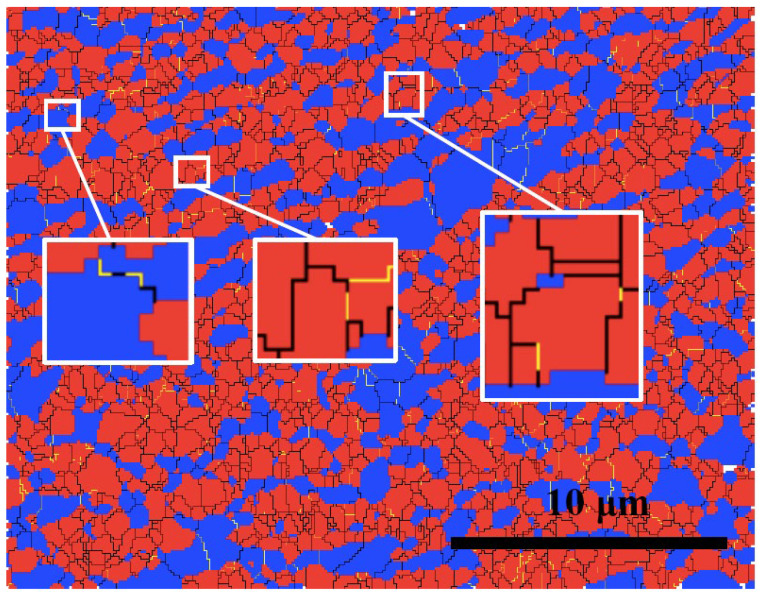
Grain boundary map of region c (LABs and HABs are in yellow and black, respectively).

The grain boundary map of region c of SZ is shown in Figure 11. There are some incomplete grain boundaries in both constituent phases, as indicated in the enlarged regions. This provides evidence of the occurrence of CDRX in the austenite and ferrite, and those LABs eventually transform into HABs [13,26].

**Figure 12:**
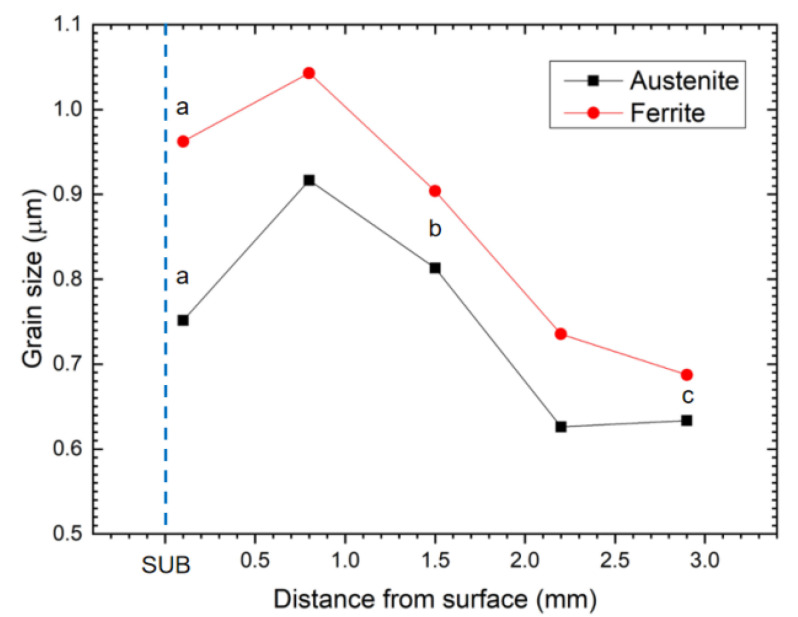
Grain size profiles of austenite and ferrite phases along the centerline. Regions a, b, and c are 0.1, 1.5, and 2.9 mm beneath the surface, respectively.

The variation in grain size of austenite and ferrite along the centerline is illustrated in Figure 12. Lakshminarayanan and Balasubramanian [36] found that greater strain and more severe plastic deformation were produced near the tool shoulder as compared to the region away from the shoulder, leading to a finer grain structure near the surface. Thus, the grain sizes of both phases show an increasing trend from the top surface (0.1 mm) to 0.8 mm. Although the strain kept on decreasing along the depth of the centerline, temperature shows a predominant influence on the microstructure [13]. The descending temperature resulted in finer grain at the bottom of the SZ (i.e., region c), which is due to the lower temperature and higher cooling rate at the bottom [28].

**Figure 13:**
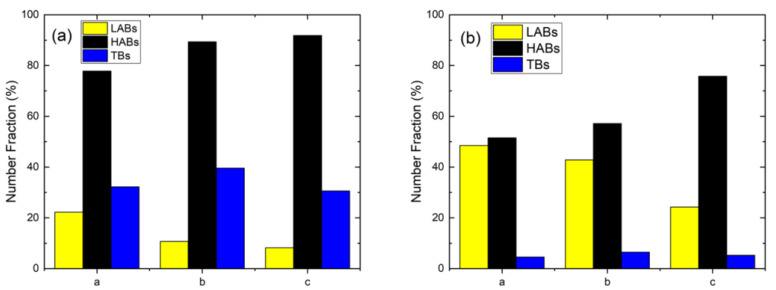
The fraction of LABs, HABs, and TBs in different regions along the centerline for FSPed SAF 2507: (**a**) γ/γ boundaries and (**b**) α/α boundaries.

From Figure 13, the fraction of HABs in the two constitute phases increases from the top to the bottom along the centerline of the SZ. As discussed before, CDRX was the dominant restoration mechanism in ferrite and austenite near the surface. However, the fraction of LABs revealed an inverse trend from the bottom towards the surface although heat generation was maximum at the plate surface [32]. It was reported that higher strain induces a larger amount of dislocations and substructures, while higher temperature accelerates the rearrangement of LABs because of the occurrence of CDRX [13]. Obviously, the strain is dominant as evidence of a decreasing trend of LABs from region a to region c.

**Figure 14:**
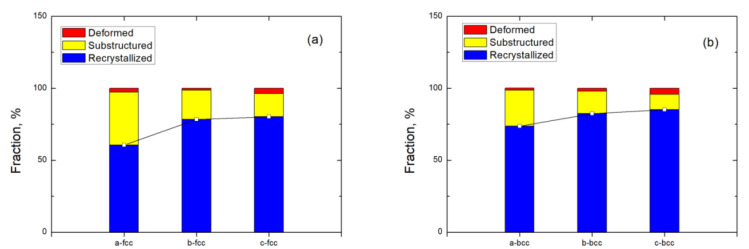
The fraction of the recrystallized, substructured, deformed grains at different regions along the centerline of FSPed specimen: (**a**) austenite and (**b**) ferrite.

Figure 14 depicts the RF maps of the FSPed specimen along the depth of the centerline. The fractions of recrystallized grains at regions b and c are almost the same, i.e., more than 80%, indicating the occurrence of complete DRX. Lower recrystallized fractions of austenite and ferrite are obtained at region a. It is attributed to the growth of the recrystallized grains after complete DRX [37], when the temperature near the surface was higher than that of the region far from the surface. Moreover, higher strain in region a would decelerate DRX in both ferrite and austenite [38]. Furthermore, the recrystallized fraction of ferrite was higher than that of austenite at the same location. This finding is consistent with the recrystallized fraction of the surface (Figure 9). It is mainly attributed to the strain accommodated by the ferrite resulting in a prior occurrence of DRX in the ferrite as compared with the austenite [38].

**Figure 15:**
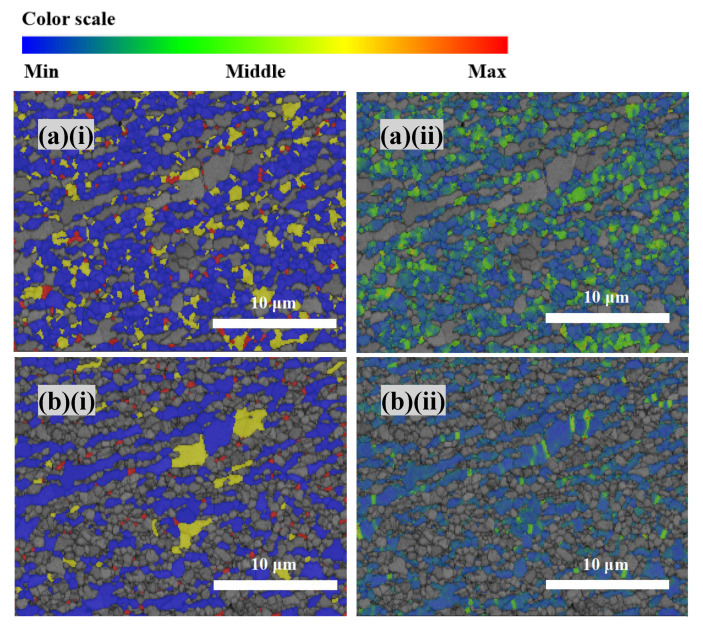
EBSD micrographs of (**a**) austenite and (**b**) ferrite showing: (**i**) RF and (**ii**) KAM maps.

The RF and KAM maps at regions a, b, and c are almost the same. Therefore, only the RF and KAM maps of region c are shown in Figure 15, which implies a big difference in microstructure developed in the austenite and ferrite. The KAM values adjacent to the grain boundary were higher than that of intragranular of the ferrite, indicating a strain concentration adjacent to the grain boundary [Figure 15b(ii)]. Kamaya and co-workers [39] reported that KAM maps could reflect the distribution of dislocation density. Consequently, the dislocations in ferrite are mainly concentrated in deformed grains and sub-boundaries, while the dislocation density is low in the subgrains and recrystallized grains. The dislocations at the grain boundaries and deformed grains provided a high diving force for nucleation of the subgrains and the DRX at the grain boundaries [37]. Furthermore, the dislocation density decreased as the recrystallized grains grew by absorbing the dislocations in the deformed grains and subgrain boundaries [33,40].

For the austenite, the dislocation density is high in deformed and substructured grains. The high strain in the austenite implies that the deformation transferred from ferrite to austenite under such strain [38]. It was reported that in the initial stage of hot deformation, strain was mainly concentrated in the ferrite. Then, the deformation transferred to austenite gave rise to form dislocations until DRX occurred under higher strain [38]. In the present study, the strain at the bottom of the SZ was high enough to transfer the load from the ferrite to the austenite and promoted the occurrence of DRX in the austenite. The strain partitioning makes the microstructure evolution of FSPed SAF 2507 more complicated.

### 3.3. Hardness

**Figure 16:**
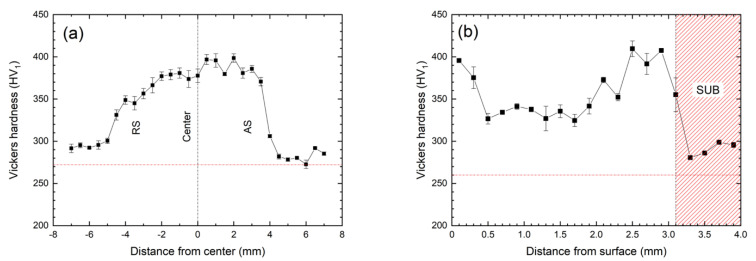
Hardness profiles of FSPed specimen in (**a**) transverse direction; and (**b**) longitudinal transverse direction (along the centerline).

The hardness profiles of the FSPed specimen in the transverse direction (0.1 mm below the top surface) and longitudinal direction (along the centerline) are shown in Figure 15. It is obvious that the measured hardness in all regions was higher than that of the substrate (272 HV_1_), as indicated by the red dash line (Figure 16). The increase in hardness of the SZ of the FSPed specimen is attributed to the generation of dislocations and the reduction in grain size [41]. According to Moshtaghi and Safyari [42], the yield stress of the specimen can be calculated by using Vickers hardness. The yield stress is proportional to the Vickers hardness. The higher the hardness value, the higher the yielding stress can be achieved. Consequently, the yield strength in all regions of the SZ was higher than that of the substrate. Cui and his co-workers [33] investigated the relationship between microstructure and hardness of FSPed AISI 201 austenitic stainless steel (ASS). It showed that the FSPed AISI 201 possessed higher hardness (260 HV) than the base material (210 HV) due to grain refinement (reduce from 30–80 mm to 4 mm) and high dislocation density in the stir zones induced by severe plastic deformation during the FSP. While the hardness in the AS and RS is similar. Moreover, Hajian and his co-worker reported that the ultrafine-grain structures (with an average grain size of 2.2 μm) were detected in the FSPed AISI 316L ASS. The hardness of the FSPed 316L (300 HV) is 1.73 times higher than that of the base material (173 HV) [43]. A sharp increase in the hardness near the upper surface of the FSPed 316L was observed (900 HV) due to the higher degree of grain refinement in this region. Moreover, the hardness of the FSPed AISI 420 martensitic stainless steel (698 HV_1_) with a grain size of 0.75 mm was reported to be dramatically enhanced as compared to the annealed 420 (184 HV_1_) with a grain size of 4 mm [18]. The difference in hardness at the AS and RS was large for the FSPed 420 fabricated at high transverse speed. The distribution of hardness of the FSPed SAF 2507 was not uniform in the traverse direction (Figure 16a). The asymmetric distribution of hardness value is due to the difference in grain sizes at AS and RS. The hardness at the AS was slightly higher than that of the RS, as the grains of both phases are finer in the AS [27].

From Figure 16b, the hardness decreases gradually near the top surface, and then it starts to increase until it reaches a peak value; finally, it decreases to the hardness of the SUB. According to the previous works [15,22,33], hardness has a close relationship with the grain size in the processed regions. According to the Hall–Petch relationship, finer grain size would result in higher hardness. Consequently, the bottom of the SZ possessed the highest hardness, indicating the finest grain size in this region. The value of hardness has a good agreement with the grain size (Figure 11) in these regions. It is worthy to notice that a high hardness (395 HV_1_) was also observed near the top surface, although the grain size was slightly larger than that of the bottom with a hardness of 407 HV_1_. As mentioned above, the strain and plastic deformation were maximum on the workpiece material closest to the tool shoulder and decreases along the depth of the SZ, resulting in higher hardness at the top surface.

## 4. Conclusions

Microstructural evolution and hardness of FSPed SAF 2507 super duplex stainless steel were investigated in the present study. The main conclusions are summarized as follows:

- Finer grain size was found in the processed zone of the FSPed SAF 2507 because of the occurrence of CDRX in both austenite and ferrite. The grain size of austenite was smaller than that of ferrite as CDRX initiated in the ferrite.
- Higher degree of grain refinement and DRX were observed at the AS of the FSPed specimens due to higher strain and temperature there. The grain size increased from the bottom to the top, as the growth of recrystallized grains occurred at a longer cooling time and a higher temperature at the top surface.
- The volume fraction of austenite increased with the decrease in temperature during FSP along the centerline from top to bottom because more ferrite is transformed to austenite at a lower temperature.
- The hardness value of SZ in different regions (360–397 HV_1_) was higher than that of the substrate (272 HV_1_) because of the generation of dislocations and the reduction in grain sizes during FSP. The generation of heat was different at the AS, center, RS, and along the longitudinal direction of the SZ, thus resulting in non-uniform hardness distribution. The maximum hardness was obtained at the bottom (407 HV_1_).

## Figures and Tables

**Table 1:** Nominal compositions of SAF 2507 SDSS (wt%).

Cr	Ni	Mo	Mn	Cu	Si	N	C	S	P	Fe
25.1	6.6	3.4	0.8	0.21	0.6	0.28	0.02	0.01	0.03	Bal.

**Table 2:** Volume fraction of austenite and ferrite in different regions.

Region	Austenite (%)	Ferrite (%)	Average Grain Size of Ferrite (μm)	Average Grain Size of Austenite (μm)
SUB	55.0 ± 3	45.0 ± 3	5.6 ± 1	6.6 ± 1
RS	54.1 ± 2	45.9 ± 2	1.5 ± 0.2	1.0 ± 0.2
Center	49.3 ± 2	50.7 ± 2	1.0 ± 0.2	0.8 ± 0.2
AS	56.9 ± 2	43.1 ± 2	0.8 ± 0.2	0.7 ± 0.2

**Table 3:** Volume fraction of austenite and ferrite in different regions.

Region	Austenite (%)	Ferrite (%)	Average Grain Size of Austenite (μm)	Average Grain Size of Ferrite (μm)
SUB	55.0 ± 3	45.0 ± 3	6.6 ± 1.0	5.6 ± 1.0
a	49.3 ± 2	50.7 ± 2	0.8 ± 0.2	1.0 ± 0.2
b	53.5 ± 2	46.5 ± 2	0.9 ± 0.2	1.1 ± 0.2
c	62.0 ± 2	38.0 ± 2	0.6 ± 0.2	0.7 ± 0.2

## Data Availability

Data sharing is not applicable for this paper.
